# Fast-track revision knee arthroplasty: A feasibility study

**DOI:** 10.3109/17453674.2011.584211

**Published:** 2011-09-02

**Authors:** Henrik Husted, Kristian S Otte, Billy B Kristensen, Henrik Kehlet

**Affiliations:** ^1^Department of Orthopedic Surgery; ^2^Department of Anesthesiology, Hvidovre University Hospital, Copenhagen; ^3^Section of Surgical Pathophysiology, Rigshospitalet, Copenhagen University; ^4^The Lundbeck Centre for Fast-track Hip and Knee Arthroplasty, Hvidovre University Hospital and Rigshospitalet, Copenhagen University, Denmark

## Abstract

**Background and purpose:**

Fast-track surgery has reduced the length of hospital stay (LOS), morbidity, and convalescence in primary hip and knee arthroplasty (TKA). We assessed whether patients undergoing revision TKA for non-septic indications might also benefit from fast-track surgery.

**Methods:**

29 patients were operated with 30 revision arthroplasties. Median age was 67 (34–84) years. All patients followed a standardized fast-track set-up designed for primary TKA. We determined the outcome regarding LOS, morbidity, mortality, and satisfaction.

**Results:**

Median LOS was 2 (1–4) days excluding 1 patient, who was transferred to another hospital for logistical reasons (10 days). None of the patients died within 3 months, and 3 patients were re-admitted (2 for suspicion of DVT, which was not found, and 1 for joint mobilization). Patient satisfaction was high.

**Interpretation:**

Patients undergoing revision TKA for non-septic reasons may be included in fast-track protocols. Outcome appears to be similar to that of primary TKA regarding LOS, morbidity, and satisfaction. Our findings call for larger confirmatory studies and studies involving other indications (revision THA, 1-stage septic revisions).

For more than a decade, favorable outcomes following fast-track protocols rather than more conventional hospital stays have been reported from numerous studies on primary THA and TKA. In the last few years, outcomes have been further improved, mainly due to improved multimodal opioid-sparing analgesia and early mobilization, allowing patients to fulfill functional discharge criteria within 2–3 days (Husted et al. 2008, 2010 a,b,c,d, Larsen et al. 2008 a,b,c, 2009, Andersen et al. 2009, Barbieri et al. 2009, Rotter et al. 2010). The addition of local infiltration analgesia (LIA) has improved early analgesia and facilitated early recovery, allowing patients to ambulate with full weight bearing within 2–3 hours of surgery (Andersen et al. 2008 a,b, 2009, Holm et al. 2010).

So far, however, no one has reported the potential benefits of the fast-track methodology (including multimodal opioid-sparing analgesia, perioperative LIA, and early mobilization) for revision TKA, with its more extensive surgical trauma leading to a corresponding increase in the surgical stress responses. We therefore investigated the feasibility of our well-documented fast-track primary TKA program on a consecutive cohort of revision TKA patients.

## Patients and methods

From January 1, 2008 until June 1, 2010, we prospectively included all patients who presented consecutively with prior TKA that required revision of both femoral and tibial prosthetic components due to aseptic loosening, wear, mechanical instability, or malposition of components. Exclusion criteria were revision surgery due to infection, simple exchange of polyethylene, and secondary operation with a patella component.

29 patients (16 women) were operated with 30 revision arthroplasties (4 rotating hinge prostheses (RHK) and 26 total condylar revision knee type prostheses (LCCK), 1 patient with bilateral simultaneous revision; all Zimmer, Warsaw, IN). Median age was 67 (34–84) years. All patients were ASA I (n = 9), II (n = 17), or III (n = 3). The operations were due to aseptic loosening, all on the tibial side (11), wear and mechanical instabilities (16), or malposition of components (3).

All patients were operated under spinal anesthesia with 3 mL 0.5% (15 mg) plain bupivacaine administered via the L2/L3 or L3/L4 vertebral interspace. Additional propofol was administered (0.5–5 mg/kg/h) for sedation if required. For thromboprophylaxis, orally administered Xarelto (10 mg) was administered once daily until discharge, starting 6–8 h postoperatively. No extended prophylaxis was given, and also no mechanical devices were used (including compression stockings). No attempt was made to identify patients at high risk of DVT, as all patients received the same regime.

A standardized intraoperative regime for fluid administration was used, consisting of 0.9% saline (5 mL/kg/h) and colloid (Voluven; 7.5 mL/kg/h). Also, a standardized program in the operating theater was followed, including use of tranexamic acid (1 g). Drains were not used. Patients were operated with insertion of a tricompartmental cemented revision prosthesis with proximal and distal uncemented stems, LCCK or RHK (Zimmer, Warsaw, IN) using a standard (extended if necessary) midline skin incision and a medial parapatellar approach (no quadriceps snips, turndowns, or tibial tubercle osteotomies were necessary). No femoral tourniquet was used. Implants were removed by using osteotomes, loosening the implants with as little sacrifice of bone as possible (the femur first, then the tibia).

The LIA technique has been described in earlier studies (Andersen et al. 2008 a,b, Kerr and Kohan 2008). Patients received infiltration with 300 mg ropivacaine (0.2%) and epinephrine (10 mg/mL) in a total volume of 150 mL. The mixture was injected using a systematic technique ensuring uniform delivery of the local anesthetic to all tissues incised, handled, or instrumented during the procedure. The first 50 mL was injected into the posterior joint capsule and into both collateral ligaments (if present and functioning) after the prosthesis had been removed and new bone cuts had been performed. After insertion of the prosthesis, another 50 mL was injected along the borders of—and into—the capsule and cut quadriceps tendon, infra-patellar ligament, possible remnants of fat pad, and soft tissues surrounding the joint. The final 50 mL was used to infiltrate the subcutaneous tissues before wound closure. To minimize the risk of cutaneous blister formation, the subcutaneous injections did not contain epinephrine.

After wound closure, a compression bandage was applied firmly from the toes to the mid-thigh, consisting of an inner double layer of soft padding surrounded by an overlapping layer of Acrylastic (elastic adhesive bandage) (Andersen et al. 2008 a). The knee was equipped with an ice pack. The compression bandage and ice pack were removed the following morning.

Postoperatively, the patients were transferred to the post-anesthesia care unit (PACU) and then to a specialized knee and hip arthroplasty unit with a well-defined and implemented multimodal fast-track rehabilitation regime (Husted et al. 2008).

Multimodal, orally administered opioid-sparing analgesia (NSAID, acetaminophen, gabapentin, and opioid upon request only) was given to all patients: celecoxib (200 mg every 12 hours), acetaminophen (slow release; 2 g every 12 hours), gabapentin (300 mg morning and 600 mg evening), all of which were initiated 2–3 hours preoperatively.

Patients left the PACU after a few hours, and attempted to ambulate upon arrival at the ward. Physiotherapy was started on the first postoperative day (after 24 hours) and took place once or twice daily until discharge. Routine exercises were performed—focusing on regaining function, motion, and gait with crutches.

LOS was counted as the number of postoperative nights in hospital until discharge. Strictly functional discharge criteria were applied (independent in personal care, able to walk > 70 m with crutches, able to get in and out of bed and into and up from a chair, and sufficient oral pain treatment (VAS < 5 on activity, and acceptance of discharge)). All patients were discharged directly to their homes.

The following outcome parameters were registered during hospitalization: LOS, duration of surgery, perioperative (anesthetic and surgical) complications, blood transfusions, and patient satisfaction—measured on a verbal analog scale (0–10; 10 = best). The patients were asked about their satisfaction with 13 parameters during their stay, including “satisfaction with LOS” and “satisfaction with the entire stay” (Husted et al. 2008 and 2010 b). A standard transfusion protocol was followed. The transfusion trigger was a drop in hemoglobin of at least 25% of the preoperative value *and* clinical symptoms of anemia.

All complications, re-admissions, or deaths within 90 days of the index arthroplasty operation were registered.

The study did not require approval by the Ethics Committee and was not registered with ClinicalTrials.gov under the US National Library of Medicine, as it was considered a quality control study.

## Results

Median surgical time was 93 (60–168) minutes. One anesthesia-related complication occurred (development of temporary second-grade AV block). 8 patients required blood transfusion (2–6 units). 7 patients had cortical cracks/fractures at the distal point of the tibial stem, and 1 patient had fracture lines at both stem ends—occurring as undetected perioperative surgical complications. At 3-month follow-up, none of the patients had progression of the fracture or complained of pain at the stem ends.

Median LOS was 2 (1–4) days but this excludes 1 patient, who was transferred to another hospital for logistical reasons (10 days).

Within 3 months, no deaths had occurred but 3 patients were re-admitted (2 for suspicion of DVT, which was not found, and 1 for mobilization of the knee). Patient satisfaction was high, median 10 (8–10), regarding both LOS and the entire stay.

## Discussion

This study shows that it is feasible to include revision TKA for non-septic reasons in a fast-track set-up and to expect an outcome similar to that of primary TKA, with low morbidity, short LOS, and a high degree of patient satisfaction (Husted et al. 2008, 2010, d). This is to be expected, as fast-track surgery consists of a combination of optimized logistics and evidence-based clinical features, thereby providing the best-documented treatment available—the “right track” (Kehlet and Søballe 2010). This fast-track set-up has optimized logistical and evidence-based clinical features and was first implemented in 2003 (Husted et al. 2008). Since then, all patients operated with THA and TKA, including bilateral simultaneous procedures, have been enrolled in the program. We have concentrated on pain treatment and early mobilization, and current research is helping to refine these tools for improvement of convalescence, thereby reducing LOS (Andersen et al. 2008 a,b and 2009, Holm et al. 2010). LIA was introduced in 2006, and has been a part of our standard fast-track protocol for primary THA and TKA ever since—and from 2008, also for revision knee arthroplasty.

We did not perform any power calculation regarding the number of revision cases needed for meaningful comparison with primary TKA, since the purpose was to determine the feasibility of using such a fast-track protocol in revision TKA compared to primary TKA. Also, comparison with a historical control group would be of little value, as our fast-track set-up is dynamic and several different aspects of it have changed over time—both clinical (anesthesia (spinal), fluid protocol, pain treatment (gabapentin, COX2 inhibitor, LIA), timing of first mobilization, thromboprophylaxis) and organizational (timing of radiographs, evaluation of discharge criteria twice daily)—compared to the early fast-track set-up (Husted et al. 2008).

Even though 8 patients had cortical fracture lines from the stem ends (indicating a press-fit that was too tight), all patients were mobilized within a few hours after surgery with full weight bearing. This rather frequent perioperative complication required no treatment or protective bracing, and did not alter the early and extensive mobilization. However, large-scale data are required to evaluate the clinical impact of this complication. LIA has been shown to facilitate early mobilization, and these revision patients were mobilized as easily and early as primary TKA patients—and with minimal or no pain for the first few hours (Andersen et al. 2008b). Early mobilization may play a key role in reducing the incidence of DVT, PE, and VTE-related death (Husted et al. 2010d). We did not encounter any of these complications; in addition, morbidity in general was low and similar to that in our fast-track results from primary TKA (Husted et al. 2010c,d).

Obstacles to be overcome in the introduction of fast-track revision surgery are limited to convincing both patients and staff that it is possible—and even desirable—to follow the same evidence-based course as in primary TKAs, despite orthopedic traditions that may dictate longer bed rest, less and lighter mobilization, and longer hospitalization. Fast-track revision TKA may bring socioeconomic benefits for both patients and society, and call for larger confirmatory studies. It might expand the indications for fast-track surgery in revision THA and 1-stage septic revisions.
